# Post-obstructive Pulmonary Oedema in Delayed Death by Hanging: A Case Report

**DOI:** 10.7759/cureus.36420

**Published:** 2023-03-20

**Authors:** Satish Kumar, Prafulla Kumar Das, Beda Nand Jha, Dhirendra Kumar Chaudhary, Sudwita Sinha

**Affiliations:** 1 Forensic Medicine, Darbhanga Medical College, Darbhanga, IND; 2 Obstetrics and Gynaecology, All India Institute of Medical Sciences, Patna, IND

**Keywords:** delayed death by hanging, suicide death, attempted suicide, post obstructive pulmonary edema, near hanging, delayed hanging death

## Abstract

Hanging is a common method to attempt suicide, which is highly lethal and results in immediate death. Near-hanging refers to those who survive a hanging injury and are able to reach a hospital. Only a few survive such episodes if they are promptly rescued, but they usually die later, which is called delayed death by hanging. Post-obstructive pulmonary oedema is a fatal complication that occurs after the removal of severe upper respiratory tract obstruction. Post-obstructive pulmonary oedema developing in delayed hanging death is not widely reported in the literature. Herein, we describe a case of post-obstructive pulmonary oedema in a 16-year-old girl with a history of attempted suicide by hanging.

## Introduction

Pressure over the neck resulting in death is a common encounter in the daily practice of a forensic specialist [1]. Medicolegally, three forms of pressure over the neck are important: hanging, manual strangulation, and ligature strangulation [1]. Hanging (self-suspension) is a common method of suicide where pressure over the neck is produced due to the weight of the body itself [1-4]. Most of the cases are suicidal, except for a few accidental cases [1]. It is a painless suicide method with immediate death due to reflex vagal inhibition, anoxia, or cerebral ischemia [1,2,3,5]. Though "once launched upon suicide by hanging, there is no retreat", a few patients survive and reach the hospital if promptly rescued, known as ‘near-hanging’ [1,3,4,5]. Those who survive usually die at some later stage termed "delayed death by hanging" [1,4]. There is a paucity of literature reporting delayed deaths by hanging, with the shortest survival period reported to be nine hours and the longest reported to be 72 days [4]. Death by hanging is caused by cerebral ischemia and anoxia due to cervical artery obstruction and/or airway obstruction leading to respiratory insufficiency or due to venous obstruction or fracture dislocation of the cervical vertebra [1,6]. Most patients develop neurological and respiratory complications immediately [3]. Delayed deaths due to hanging occur due to infarction of the brain, hypoxic encephalopathy, aspiration pneumonia, infections, Duret haemorrhage, oedema of the lungs, oedema of the larynx, or pulmonary complications like acute respiratory distress syndrome (ARDS) [4,5]. Post-obstructive pulmonary oedema (POPE), which is a fatal complication, occurs after acute and severe upper respiratory tract obstruction removal [5]. Although post-obstructive pulmonary oedema after several cases of upper airway obstruction like laryngospasm during intubation or after anaesthesia in the postoperative period has been described, post-obstructive pulmonary oedema in delayed death by hanging has not been reported widely in the literature, with less than 20 cases reported till date [5]. It is a form of "non-neurogenic, non-cardiogenic" pulmonary oedema that rapidly develops, without warning, within minutes following relief of severe upper airway obstruction (occlusion or narrowing of the airway leading to compromise in ventilation) [5]. It was described for the first time in 1973 [5]. There are two subclasses: type one is due to forced inspiration in severe and acute upper respiratory tract obstruction and in cases of drowning; type two occurs on relieving a chronic partial respiratory tract obstruction like hypertrophied adenoids or tonsils [5]. The exact mechanism of POPE development in delayed death by hanging is still not clear [5]. One theory suggests that hypoxia of the brain in hanging results in the release of vasoactive mediators like serotonin, kinins, and histamine, which results in pulmonary hypertension, pulmonary vasoconstriction, and pulmonary congestion [3,5]. Another theory postulates that there is damage to the pulmonary capillary membrane, resulting in increased capillary permeability, thereby pulmonary oedema [3,5]. The third theory postulates that sudden relief of acute upper airway obstruction causes an abrupt fall in intrapulmonary pressure, which leads to a sudden increase in venous return and increases pulmonary hyperaemia [3,5].

## Case presentation

A case of post-obstructive pulmonary oedema leading to delayed death by hanging is described in this report. A 16-year-old unmarried girl was brought to a private hospital with an alleged history of attempting suicide by hanging with her dupatta (a scarf) around her neck. She was rescued by her parents, who removed the ligature from her neck and brought her down from suspension immediately. Thus, the duration of the hanging was unknown as the event was unwitnessed. The girl was last known to have been seen 30 minutes before she was found. The time to reach the hospital was around 60 minutes. At the time of the presentation, she was unconscious and unresponsive, with a Glasgow Coma Scale (GCS) of 6/15. There was froth coming from the mouth, congestion of the face, and bluish discolouration of the hands and legs. A partial ligature mark was seen around the neck. Pupils were dilated bilaterally and sluggishly reacting to light. Her respiratory rate was increased to 48 breaths per minute, and saturation on pulse oximetry was 85%. The heart rate was 130 beats per minute, and the blood pressure was 86/42 mmHg. On auscultation of the chest, crepitations were heard bilaterally. Resuscitative measures were initiated. Intensive therapy was undertaken to improve tissue oxygenation, reduce intracranial pressure (ICP), and prevent neurological consequences. Intravenous access was secured, and fluids, cerebral decongestants, prophylactic parenteral antibiotics, and anti-epileptics were administered. Endotracheal intubation was done to secure her airway. Mechanical ventilation was provided. A few hours later, her respiratory and neurological conditions worsened, with pink frothy secretions filling the endotracheal tube despite repeated suctioning, consistent with pulmonary oedema. A tracheostomy was done to clean and remove secretions from the airway. Despite all resuscitative efforts, she developed cardio-respiratory arrest, leading to her death.

**Figure 1:**
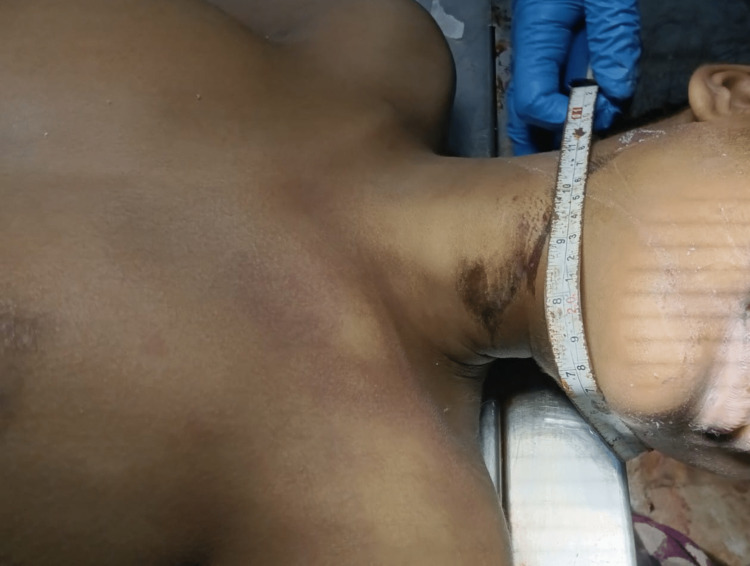
The ligature mark on the neck

She died after 24 hours of suspension. A medicolegal autopsy was ordered. The dead body was brought for medicolegal postmortem examination at the mortuary at the Department of Forensic Medicine and Toxicology (FMT), Darbhanga Medical College (DMC), Laheriasarai, Darbhanga. On external examination, a ligature mark was found that was oblique and non-continuous on the upper part of the neck above the thyroid cartilage in a 13’’ by 1.5’ dimension going upward and backwards along the lower jaw up to the right mastoid region where it was found absent as shown in Figure 1. Here an irregular compression with an inverted V, i.e., a knot mark, was seen in a 1.5’ by 2/3’ area. Evidence of a therapeutic tracheostomy was present. Subcutaneous and intramuscular haemorrhages in the neck were not observed. On autopsy examination, she was found to be five feet, two inches tall and thin. The base of the ligature mark was dark brown, hard and leathery, and parchment-like with excoriation.

**Figure 2:**
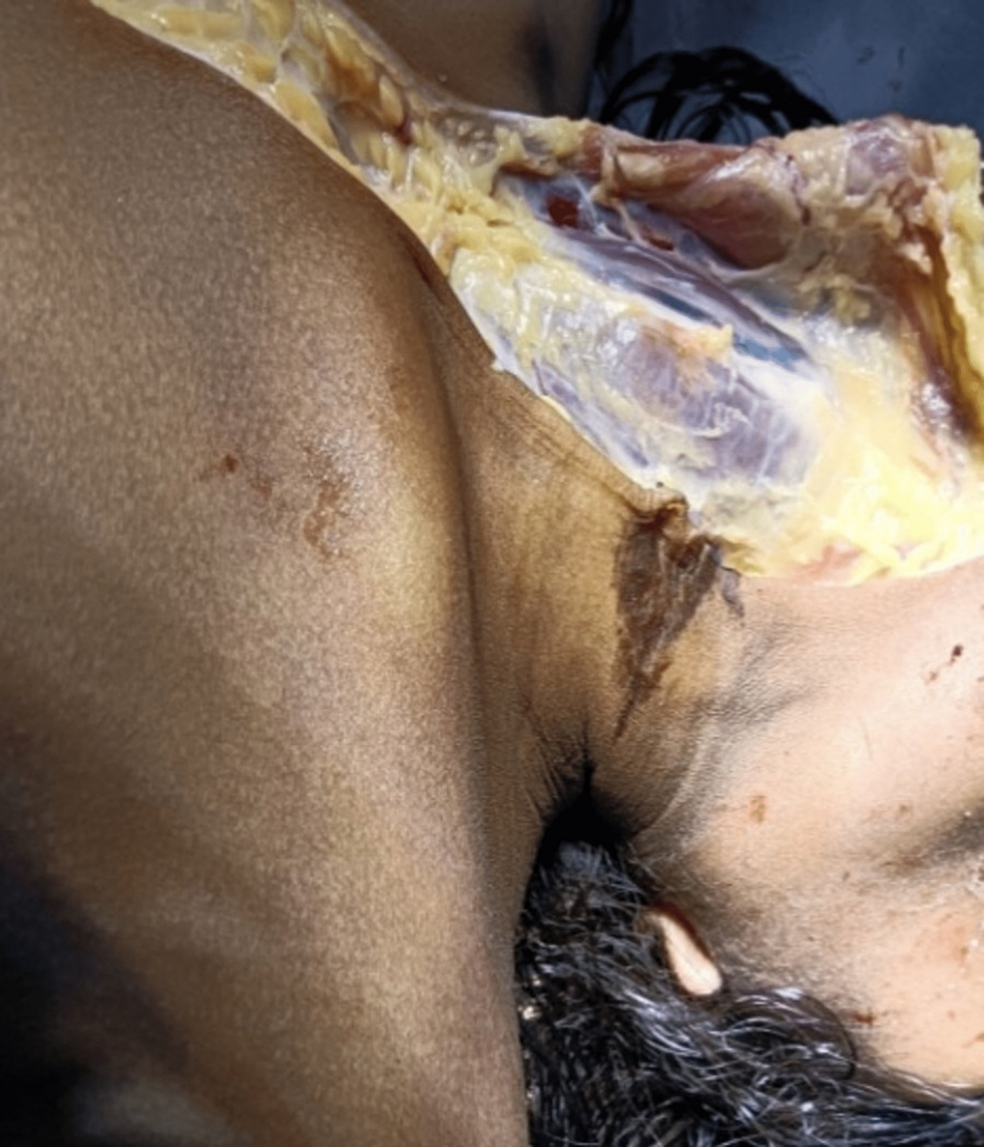
Autopsy findings of the neck

The underlying subcutaneous tissue was white and glistening, as shown in Figure 2. Neck muscles were mildly congested. The larynx and trachea were oedematous and deeply congested. The right side of the heart was full of dark blood, and the left side was empty.

**Figure 3:**
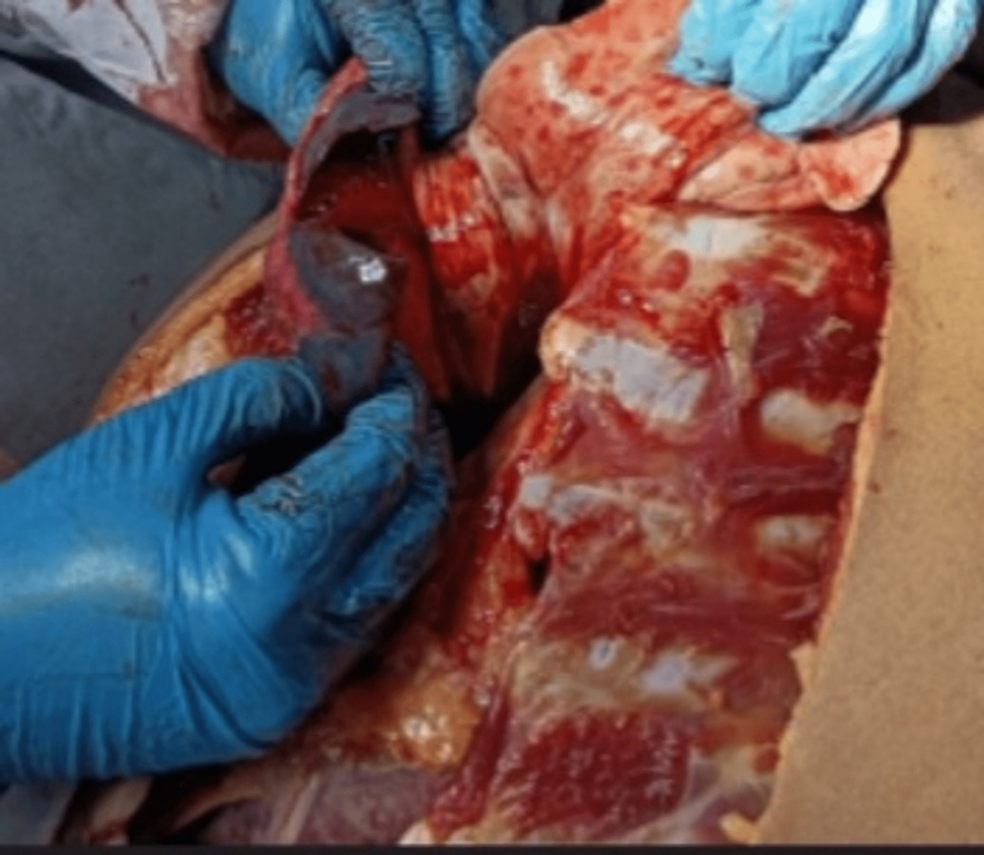
Findings of the lungs upon internal examination

On internal examination, both lungs were deeply congested and edematous, especially in the lower lobes, which appeared haemorrhagic as shown in Figure 3.

The brain and its meninges were mildly congested. Diffuse pulmonary oedema findings were confirmed on autopsy examination. An autopsy was conducted, which confirmed the diffuse pulmonary oedema. Death was due to asphyxia, oedema of the larynx and lungs, and aspiration pneumonia as a result of hanging and its secondary complications. The forensic team concluded that "complications following hanging" were the cause of death. The manner of death was suicide.

## Discussion

Survival in near-hanging is determined by many factors like the duration of the suspension, early resuscitation measures, the constricting force applied, the drop force, the point on the neck where suspension is applied, whether hanging is total with suspension in the air, the time when compression around the neck was released, narcotic and drunken states, children and the elderly, physical infirmity, other co-morbidities associated like cardiorespiratory ailment, the incomplete encirclement of the neck by the ligature, and partial hanging [4]. There are various methods of hanging, such as self-suspension to a low point, which is convenient, or to a high point [1]. Materials may include ropes, strings, wires, cloth, neckties, scarves, etc. [1]. In the above case, there was no laryngeal fracture, fracture of the hyoid bone, or haemorrhage of soft tissue because the ligature material used, "dupatta" (a scarf), was soft. Death is immediate in most hanging cases as a result of reflex vagal inhibition or cerebral ischemia and anoxia, but in the above case, death was delayed, which is uncommon although such cases of delayed death have been reported in the literature [5]. In near-hanging patients, several complications are known to occur, ultimately resulting in delayed death in these patients. These complications may include hypoxic or ischemic brain damage, intracranial haemorrhage or infarction, secondary cerebral injury, Duret haemorrhage, pulmonary complications, infections, acute respiratory distress syndrome, etc. [2,5]. Kazuhiko Kibayashi et al. reported a case of delayed death by hanging due to traumatic dissection of the carotid arteries, in which the patient survived for two and a half months before succumbing to death caused by aspiration pneumonia [6]. MR Sane et al. described 10 cases of delayed deaths by hanging with survival periods ranging from nine hours to 72 days, and the common causes of deaths were hypoxic encephalopathy and pneumonia [4]. Aggarwal et al. reported a case of delayed death by hanging in which the patient survived for nine days and finally succumbed to death due to cerebral ischemia [7]. Kanchan and Atreya reported a case of delayed death by hanging in which the patient survived for eight days and then developed cerebral oedema leading to transtentorial herniation resulting in brainstem haemorrhage (Duret haemorrhage) causing death [8].

A few cases of post-obstructive pulmonary oedema leading to delayed death by hanging have also been reported in the literature. Several factors contribute to the development of post-obstructive pulmonary oedema, also known as negative pressure pulmonary oedema. Forced inspiratory efforts against a closed upper respiratory tract result in considerable negative transpulmonary pressure, which is the initiating factor [5]. This results in increased blood flow to the right atrium and ventricle due to an increase in venous return [5]. This increased pressure is transmitted to the pulmonary alveoli and interstitium, leading to an increase in hydrostatic pressure resulting in the transudation of fluid across the pulmonary membrane from the capillaries to the interstitial space [5]. All these factors, combined with the hyperadrenergic state and hypoxic state (causing increased pulmonary capillary pressure and pulmonary vascular resistance due to the redistribution of blood from systemic to pulmonary vasculature), contribute to the development of pulmonary oedema [5]. Similar to this case, MA Mesrati et al. reported a case of delayed death by hanging due to post-obstructive pulmonary oedema in a 10-year-old girl, in which the patient had survived for three days [5]. Mahendra Kumar et al. reported a case of near-hanging during an attempted suicide in a 25-year-old male who developed pulmonary oedema but was resuscitated and treated successfully [3]. The patient was rescued by relatives within a few minutes and shifted to the hospital within 30 minutes, whereas in the above-presented case, the duration of the hanging was not known, and after being found, the time to reach the hospital was about 60 minutes. Moreover, the patient had no features of pulmonary oedema at presentation but developed them after two hours, whereas in the above-presented case, the patient had features of pulmonary oedema at the time of hospital presentation. These are two important factors that determine the patient's survival chances and thus result in successful resuscitation [3].

## Conclusions

The fatal period is not well determined in hanging and may range from minutes to days to weeks or even months. It may depend on factors such as the constricting force used, the time taken for the patient to be found and relieved of neck compression, the time taken to reach the hospital, the resuscitative measures used, etc. Patients who survive hanging should never be considered invulnerable. Although hypoxic-ischemic encephalopathy is the most common reason for mortality in delayed suicidal hanging death cases, post-obstructive pulmonary oedema should be considered a potentially lethal complication in such patients.
